# Supplementary figures and images for: Comparative phosphoproteome analysis to identify candidate phosphoproteins involved in blue light-induced brown film formation in Lentinula edodes
Source: PeerJ. 2020 Dec 18;8:e9859. doi: 10.7717/peerj.9859 (PMC7751435; doi:10.7717/peerj.9859)

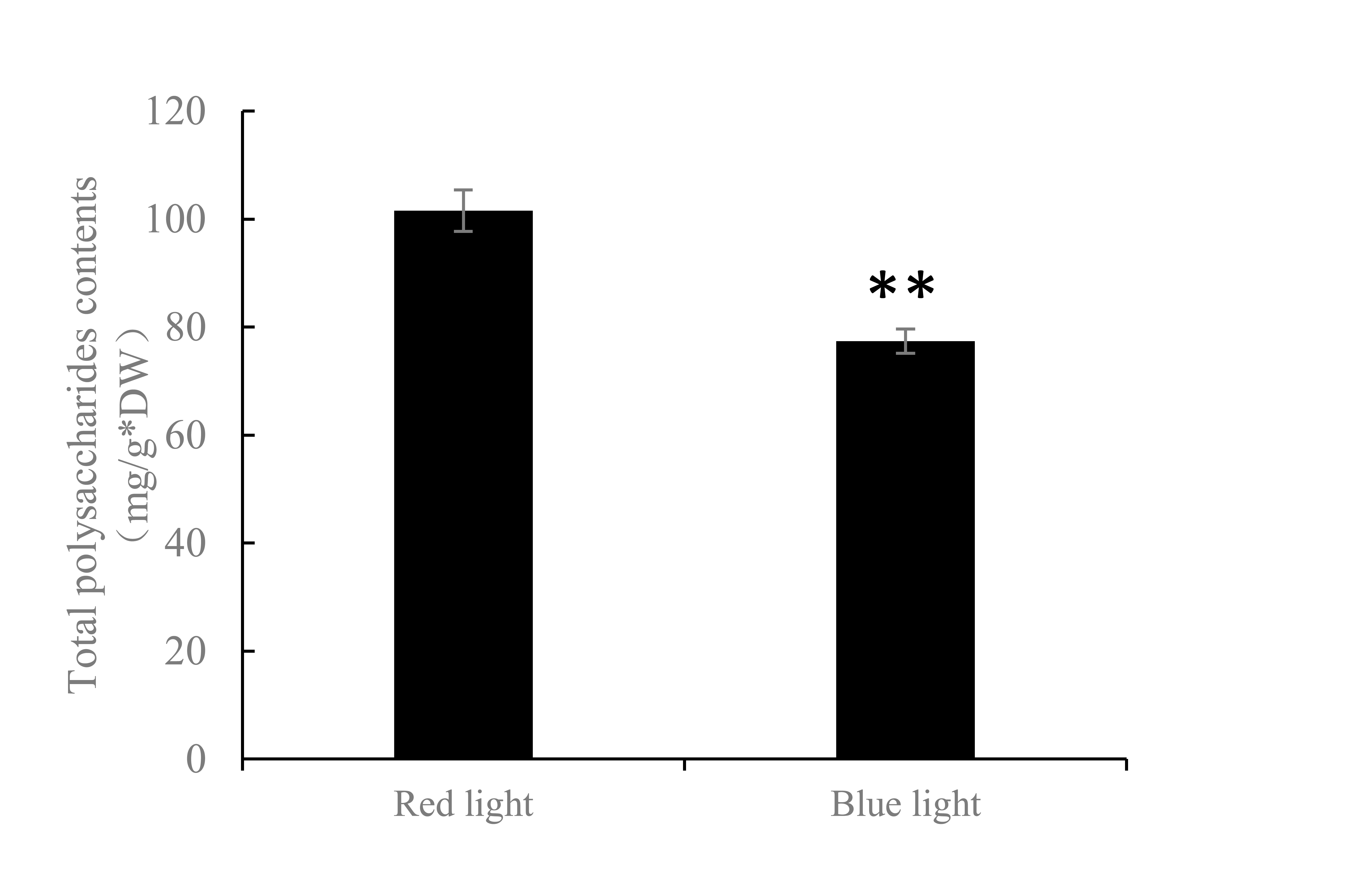

Supplement: Figure S1 [file peerj-08-9859-s006.png]
